# Scalpel blade contamination and risk of postoperative surgical site infection following abdominal incisions in dogs

**DOI:** 10.1186/s13104-019-4494-7

**Published:** 2019-07-25

**Authors:** Christina G. Lioce, Elizabeth C. Davis, Julie W. Bennett, Forrest I. Townsend, Christopher P. Bloch

**Affiliations:** 1 Surgery Department, New England Animal Medical Center, West Bridgewater, MA USA; 2 Department of Biological Sciences, Bridgewater State University, Bridgewater, MA USA

**Keywords:** Scalpel, Contamination, Surgical site infection

## Abstract

**Objective:**

This prospective observation sought to determine if scalpel blades used for abdominal skin incisions in dogs are a significant source of bacterial contamination, and if these blades should be changed prior to use in deeper dissection.

**Results:**

Scalpel blades were swabbed for culture prior to skin incision as a control, and then again following ventral midline abdominal skin incision in a total of 75 dogs. Culture and sensitivity results were compared with review of medical records for any evidence of pre- or postoperative incisional surgical site infection/inflammation (SSI). Of the 75 blades swabbed after skin incision, only 2 (2.7%) had positive culture results. Of the 69 patients that survived to suture removal, there was evidence of SSI in 6 patients (8.7%), only one of which had a positive scalpel blade culture (16.7%). Neither the use of postoperative antibiotics nor positive scalpel blade culture results were good predictors of whether a patient would develop a SSI. Results of this pilot study suggest that there is no bacteriological evidence to support the use of a separate blade for deep dissection in routine surgical procedures.

**Electronic supplementary material:**

The online version of this article (10.1186/s13104-019-4494-7) contains supplementary material, which is available to authorized users.

## Introduction

Postoperative infections are an inherent risk of any surgical procedure despite preventative measures. Previously published rates of surgical site infections (SSI) in small animals have ranged from 3–10% [1–4]. Risk factors associated with SSI include duration of surgery, gender, increasing body weight, dirty surgical site, antimicrobial prophylaxis, and use of propofol [1, 4]. Straw et al. [5] described that up to 20% of skin bacteria is not susceptible to disinfection, being a potential source of bacterial contamination leading to incisional infections.

Numerous human medical studies have implied that use of separate blades as a means of infection control is unnecessary [6–11]. However, surgical wound contamination and infection statistics from human patients may not serve as a correlate to canine surgery. To the author’s knowledge, there is only one report in the veterinary literature that examined scalpel blade contamination with skin bacteria, which only included orthopedic and neurosurgical procedures in dogs but found that the skin blade does not add significantly to bacterial inoculum contaminating clean wounds [5]. While deep tissue contamination can have a tremendous impact on orthopedic and neurosurgical procedures, there is currently no information on scalpel blade contamination from ventral midline abdominal incisions in dogs. Therefore, evaluating risk of SSI in this common surgery is warranted, and adds evidence to previous reports.

The purpose of this study was to determine if scalpel blades used for ventral midline abdominal skin incisions are a significant source of bacterial contamination for deeper structures in dogs, and if they should be changed prior to deeper dissection. We hypothesized that there would be little to no contamination of the scalpel blades used for skin incision, which would also not correlate with postoperative SSIs, and therefore in order to prevent infection the scalpel blade does not need to be changed prior to deeper dissection.

## Main text

### Materials and methods

An informal survey of 43 veterinary surgeons was undertaken to evaluate incidence of changing blades intraoperatively and included reasons for changing blades. The survey was performed using an anonymous survey website (SurveyMonkey Inc, San Mateo, CA).

Canine patients undergoing ventral midline abdominal incisions longer than 10 cm were included in the study, regardless of procedure performed. Patients that did not survive to suture removal or 30 days postoperatively were excluded from analysis of SSI risks. Each patient was aseptically prepared using 2% chlorhexidine gluconate scrub and 70% isopropyl alcohol according to standard recommended protocol [12]. The environmental conditions in the surgical suite were similar for each case. Each patient also received a prophylactic dose of either cefazolin 22 mg/kg IV or ampicillin/sulbactam 50 mg/kg IV within 30 min prior to skin incision.

Two samples were obtained from each patient. A sterile #10 blade was placed on the instrument table, then swabbed with a sterile moistened culturette as the control sample prior to contact with skin. The surgical area was then isolated with four cloth drapes and overlying fenestrated drape, and the ventral midline abdominal incision was made with the #10 blade. Immediately after skin incision, the #10 scalpel blade was swabbed with another sterile moistened culturette as the study sample. The surgeon’s assistant performed this sampling sterilely before contact with the patient’s skin, and sampling of each side and along the edge of each blade (all that contacted the skin during incision) was performed. Each surgical procedure progressed accordingly, without changing instruments, gloves, or blades after sampling.

The microbiological sampling technique and testing was similar to that used in previous veterinary studies [13–15]. Submitted swabs were stored in Amies Clear gel preservation medium with sodium thioglycolate and planted on a Trypticase Soy Agar w/ 5% sheep blood and a MacConkey agar plate. The culture plates were examined for growth at 24 and 48 h, and if present the species was identified. If needed, a subculture was performed to blood agar and Mac agar respectively to isolate individual bacterial colonies. The plates were then held an additional 24 h. If no changes were noted, the identification and quantity were reported out as final.

Data was collected from medical records and follow-up calls to primary veterinarians if needed, as detailed in Table 1. Inclusion criteria for evaluation of postoperative SSI were survival to suture removal, a positive bacterial culture of the incision, or a diagnosis and antimicrobial treatment by a veterinarian after visual assessment of purulent discharge, heat, redness, pain, or localized swelling of the incision. Patients were excluded from evaluation of postoperative SSI if they died or were euthanized prior to suture removal and not for reasons related to SSI, and if medical records or contact with their primary veterinarian did not provide information regarding incisional healing within 30 days postoperatively.

**Table 1:** Data variables collected for each patient and definitions of their reporting

Variable	Definition
Patient signalment	Age, sex, breed, weight
Procedure performed	Clean vs. clean-contaminated vs. contaminated vs. dirty
Duration of anesthesia	Time from induction to cessation of isoflurane administration, in minutes
Perioperative antibiotic	Cefazolin 22 mg/kg IV or ampicillin/sulbactam 50 mg/kg IV
Propofol	Used vs. not used, 4–6 mg/kg IV
Preoperative skin condition	Within normal limits vs. scrub irritation vs. previous incision (within 30 days) vs. pyoderma vs. dirty
Incision length	Greater than or equal to 10 cm
Control and study sample culture identifications	Positive vs. negative, isolate
Postoperative evidence of SSI	As defined by the Center for Disease Control (CDC, Table 2)

**Table 2:** CDC criteria for defining surgical site infection [4]

	Superficial incisional SSI	Deep incisional SSI	Organ/space SSI
Timing	Within 30 days of surgery	Within 30 days of surgery or 1 year if implant in place	Within 30 days of surgery or 1 year of implant in place
Location	Only skin or subcutaneous tissues of incision	Deep soft tissues (fascia, muscle) of the incision	Any area other than the incision which was opened or manipulated in surgery
Clinical aspects^a^	Purulent discharge Organisms isolated from an aseptically collected sample of fluid or tissue One or more: pain/tenderness, localized swelling, redness, heat, and incision is deliberately opened by surgeon unless culture negative	Purulent drainage from deep incision but not organ/space Deep incision spontaneously dehisces or is deliberately opened when patient has one or more: fever, localized pain/tenderness unless culture negative Abscess or other evidence of infection on direct exam, during reoperative, or by histopathology or radiology	Purulent drainage from drain placed in organ/space Organisms isolated from aseptically collected sample from organ/space Abscess or other evidence of infection on direct exam, during reoperation or by histopathology or radiology Diagnosis of organ/space SSI by attending clinician

^a^One or more must be present

Statistical analyses were conducted using SPSS v.24 software. Significance was assessed at the level of α = 0.05. Logistic regression was used to model the effects of continuous variables (age, weight, anesthesia time, incision length) on the likelihood of a SSI. Fisher’s Exact Test was used to evaluate associations between the frequencies of discrete variables (counts) and the frequency of SSI.

### Results

**Fig. 1:**
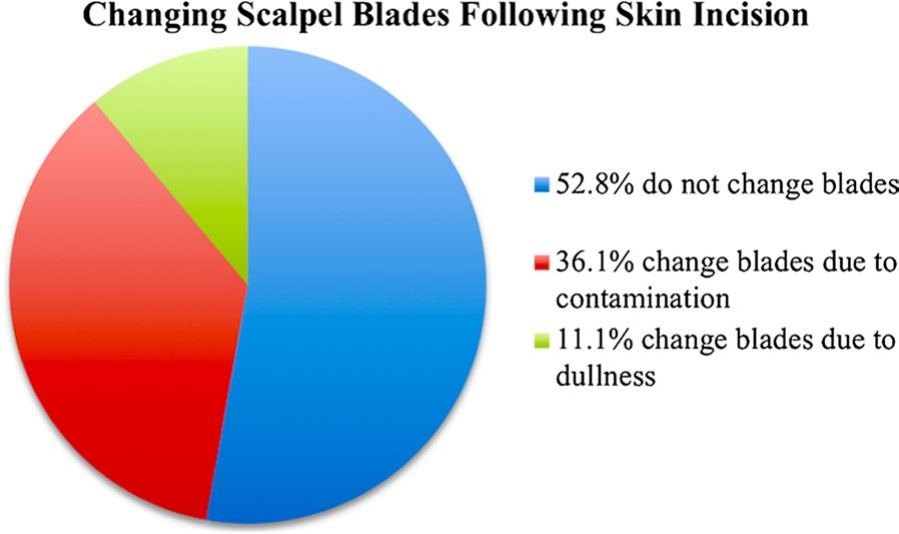
Informal anonymous survey results regarding whether or not, and why, board-certified veterinary surgeons currently change scalpel blades following skin incision and before deeper dissection

Results of the informal survey of veterinary surgeons revealed that 36.1% of them change their scalpel blades after initial skin incision, as they feel the skin blade could potentially introduce bacteria into the deeper surgical field (Fig. 1).

A total of 75 dogs that underwent ventral midline abdominal incisions were eligible for inclusion in the study. Of the 75 scalpel blades cultured after skin incision, there were only 2 that were positive for bacterial growth (2.7%). One sample was positive for *Acinetobacter *sp., and that patient did not develop a postoperative SSI. The second positive blade culture grew *Staphylococcus pseudintermedius*; that patient had an erythemic previous spay incision present at the time of surgery and also went on to develop incisional erythema and purulent discharge. Unfortunately, the owners declined sampling the incision, so the presumed SSI was not confirmed via culture.

There was one positive control sample, which was a suspected laboratory/testing contaminant (*Achromobacter *sp.) rather than a true skin incision contaminant. Six dogs (8.0%) died or were euthanized prior to suture removal for reasons unrelated to SSI (poor prognosis, decompensation). Of the 69 dogs that survived to suture removal, six (8.7%) reportedly developed and were treated for a SSI but only two (2.9%) were confirmed with positive culture results. One of those incisions was infected with *Eschericia coli* and *Enteroccocus *sp., and the other infected with *Eschericia coli* and *Streptococcus *sp. One of the diagnosed SSIs had a negative culture despite the presence of purulent discharge, and three did not have cultures performed as they were declined by the owners. All diagnosed SSIs were treated successfully with cephalexin, cefpodoxime, or amoxicillin/clavulanic acid.

Preoperative skin condition was recorded for every patient, and abnormalities were noted in 15 patients (20%). Abnormalities noted were scrub irritation (8 cases, 10.7%), previous healing or healed incision within 30 days postoperatively (2 cases, 2.7%), pyoderma (2 cases, 2.7%), and visible dirt/debris (3 cases, 4.0%). Preoperative skin condition was a poor predictor of whether a patient would experience a SSI. All four continuous variables were poor predictors of whether a patient would experience a SSI (Additional file 1: Table S1). None of the regression models were statistically significant (P > 0.05), and model fit was poor ( < 0.15) in all cases. There was no significant association between the frequency of SSI and any of the observed discrete variables (P > 0.05 in all cases; Additional file 1 Table S2).

### Discussion

Bacterial contamination rate of scalpel blades used for ventral midline abdominal incision in dogs has not been previously reported. In the present study, only 2.7% of scalpel blades had positive cultures after incising through skin. The use of a chlorhexidine neutralization step was not included in microbiological testing, which may have significantly increased the number of false negative cultures. However, the purpose of this study was to evaluate the potential risk of contamination following a standard skin preparation protocol, which would not include a chlorhexidine neutralization step prior to further scalpel blade use after skin incision. Therefore, our results reflect the true clinical situation of abdominal surgical procedures.

The bacterial species isolated from the two positive scalpel blade cultures were *Acinetobacter *sp. and *Staphylococcus pseudintermedius,* both of which are common opportunistic skin pathogens of concern due to their high level of antimicrobial resistance [4]. Despite this concern, only one of those two dogs whose scalpel blades were positive for bacterial growth went on to develop a presumed SSI, which was unfortunately not confirmed via culture but responded to treatment with amoxicillin/clavulanic acid. This patient’s blade cultured *Staphylococcus pseudintermedius*, and this dog had undergone a routine spay by their primary care veterinarian within 30 days of their surgery included in this study. On initial presentation, this previous spay scar already appeared subjectively inflamed, likely resulting in the positive bacterial culture of the scalpel blade used to make the abdominal incision over the scar. Based on these results, the scalpel blade used to incise skin during abdominal surgery is likely not a significant source of contamination but there may be increased risk of SSI if there is already evidence of inflammation or infection. Whether or not the skin scalpel blade is contaminated is not a good predictor of whether a patient will suffer a SSI, but if incising through active infection or SSI the scalpel blade likely should be changed and the affected tissue cultured or even excised prior to closure.

In the present study, there were six (8.7%) postoperative SSIs diagnosed, which is higher than reported in more recent studies of SSI rates in small animal surgery. Despite previously reported rates of 3–10%, a study by Turk et al*.* found that in 846 dogs undergoing various surgical procedures there were only 26 (3.0%) identified SSIs [2]. They also found that hypotension, class of surgery, and use of an implant increased risk of SSI. A more recent large study of 1271 dogs and cats reported a low and very similar rate of SSI at 2.83% [16]. Further study of scalpel blade contamination and SSI risk with a larger number of cases is warranted.

### Conclusions

Regardless of the level of care provided, postoperative infections are an inherent risk of surgery and will likely continue to be a source of investigation. The present study showed very low bacterial contamination on skin scalpel blades, however was underpowered to evaluate relationship to SSI.

## Limitations

There were several limitations of the present study. Most notable was the relatively small sample size, leading to only two positive scalpel blade cultures (2.7%) and six postoperative SSIs (8.7%). This prevented any strong correlation of previously reported significant factors in development of SSI. Although logistic regression models correctly predicted presence or absence of an SSI in most cases, this was because each model predicted no SSI for every case, while SSIs were uncommon (6 of the 69 cases in which the patient survived long enough to assess). Power of analysis calculations showed that definitive statements regarding the results of this study require 10 each of positive scalpel blade cultures and diagnosed SSIs, however this study proves the low incidence of positive scalpel blade cultures out of 75 abdominal incisions and can preliminarily report their lack of significance in risk of postoperative SSI.

Another limitation of this study was that of the SSIs diagnosed by different veterinarians based on the CDC’s definitions, only half actually had cultures performed at the time of diagnosis, and one of those cultures returned negative for bacterial growth despite purulent discharge being present. All of the diagnosed SSIs were treated successfully with antibiotics, empirically or based on culture results, but confirmation with a positive culture of the incision would have been ideal. Finally, a different method of scalpel blade sampling and testing (e.g., immersion in thioglycollate broth or sonication) for culture could potentially yield higher bacterial counts and different results. However, as stated before, swabbing of the scalpel blade areas that came in contact with the skin during incision was sufficient for the purposes of this study.

## Additional file

**Additional file 1: Table S1.** Results of logistic regression analysis used to model the effects of continuous variables (age, weight, anesthesia time, incision length) on the likelihood of a SSI^1^. **Table S2.** Results of Fisher’s Exact Tests to evaluate associations between SSI frequency and discrete variables.

## Data Availability

The datasets used and/or analyzed during the current study are available from the corresponding author on reasonable request.

## Abbreviation

SSI: surgical site infection
