# Supplementary material for: Scalpel blade contamination and risk of postoperative surgical site infection following abdominal incisions in dogs
Source: BMC Res Notes. 2019 Jul 25;12:459. doi: 10.1186/s13104-019-4494-7 (PMC6659296; doi:10.1186/s13104-019-4494-7)
Supplement: Supplementary file 1 — Additional file 1: Table S1. Results of logistic regression analysis used to model the effects of continuous variables (age, weight, anesthesia time, incision length) on the likelihood of a SSI1. Table S2. Results of Fisher’s Exact Tests to evaluate associations between SSI frequency and discrete variables. [file 13104_2019_4494_MOESM1_ESM.docx]

**Table S1. Results of logistic regression analysis used to model the effects of continuous variables (age, weight, anesthesia time, incision length) on the likelihood of a SSI^1^.**

| **Independent variable** | **χ^2^** | **P** | **R^2^** | **β** | **Classification** |
| --- | --- | --- | --- | --- | --- |
| Age (months) | 1.29 | 0.255 | 0.05 | -0.01 | 0.91 |
| Weight (kg) | 0.72 | 0.397 | 0.02 | 0.03 | 0.91 |
| Anesthesia time (min) | 0.04 | 0.841 | <0.01 | 0.00 | 0.91 |
| Incision length (cm) | 2.62 | 0.106 | 0.11 | -0.25 | 0.90 |

^1^ The test statistic (Wald χ^2^) is considered significant if P < 0.05. Nagelkerke R^2^ is analogous to the coefficient of determination of ordinary least squares regression. β represents the slope of the regression model, and can be interpreted based on odds ratios (β = 2.0 indicates a 2-fold increase in the odds). Classification is the proportion of observed cases for which the presence or absence of SSI would have been correctly predicted by the regression model. The classification proportion for incision length differs from the others only because the sample size differed (i.e., incision length was not measured for 6 patients).

**Table S2. Results of Fisher’s Exact Tests to evaluate associations between SSI frequency and discrete variables.**

| **Independent variable** | **P** |
| --- | --- |
| Sex | >0.999 |
| Intraoperative antibiotic | 0.656 |
| Propofol | >0.999 |
| Preoperative skin condition | 0.072 |
| Skin staples | 0.167 |
| Postoperative antibiotic | >0.999 |
| Scalpel blade culture results | 0.170 |

P represents 2-tailed probabilities.
